# Curcumin suppresses the progression of gastric cancer by regulating circ_0056618/miR-194-5p axis

**DOI:** 10.1515/biol-2021-0092

**Published:** 2021-09-06

**Authors:** Shan Li, Lihai Zhang, Shuhua Li, Hengyi Zhao, Yonggang Chen

**Affiliations:** Department of Medicine, Qilu Hospital (Qingdao), Cheeloo College of Medicine, Shandong University, Qingdao, China; Department of General Surgery, The First Affiliated Hospital of Jiamusi University, Jiamusi, China; Department of Drug, Medical Apparatus Supply, Zhongyuan Oilfield General Hospital, Puyang, China; Department of Clinical Pharmacy, Xuzhou Central Hospital, No. 199, Jiefang South Road, Xuzhou 221009, China

**Keywords:** gastric cancer, curcumin, circ_0056618, miR-194-5p

## Abstract

Curcumin has been demonstrated to be an anti-tumor agent in many types of cancers, including gastric cancer (GC). However, the molecular mechanisms by which curcumin performs its anti-tumor effects remain elusive. circ_0056618 and miR-194-5p are reported to be involved in GC progression, but their relationships with curcumin are unclear. In this study, circ_0056618 was elevated, and miR-194-5p was reduced in GC tissues and cells. Curcumin treatment led to a decrease in circ_0056618 level in GC cells. Overexpression of circ_0056618 promoted cell proliferation, migration, and invasion and suppressed cell cycle arrest and apoptosis in curcumin-treated GC cells. Moreover, miR-194-5p was identified as the target of circ_0056618, and its expression in GC cells increased after curcumin treatment. Overexpression of miR-194-5p reversed the promotional effect of circ_0056618 on cell progression in curcumin-treated GC cells. Additionally, curcumin treatment repressed the tumorigenesis of GC *in vivo* through regulating circ_0056618. Curcumin treatment delayed the development of GC partly through decreasing circ_0056618 and increasing miR-194-5p.

## 1 Introduction

As a cancer with high incidence worldwide, gastric cancer (GC) severely affects the quality of people’s life [1,2]. Presently, the primary therapeutic strategies for GC include surgery, chemotherapy, and targeted therapy [3]. Unfortunately, although diagnosis and therapy methods have been improved, the prognosis remains poor due to cancer relapse and metastasis [4,5]. Thus, there is an urgent need to search for novel treatment methods for this lethal disease.

Curcumin is a natural polyphenolic compound isolated from turmeric, which has lipid-lowering, anti-tumor, anti-inflammation, and anti-oxidation effects [6,7]. Recently, studies have shown that curcumin can reduce the malignancy of several cancers, such as pancreatic cancer [8], retinoblastoma [9], osteosarcoma [10], and bladder cancer [11]. Moreover, curcumin has also been reported to have an anti-tumor effect on GC [12,13]. Nonetheless, the role and underlying mechanism of curcumin in GC have not been well recognized.

circular RNAs (circRNAs) are a family of non-coding RNAs (ncRNAs) that possess closed-loop structures and play a vital role in gene expression via competitive targeting microRNAs (miRNAs) [14]. circRNAs have been verified to play essential roles in the carcinogenesis of GC. For example, circ_104916 could hamper the viability and metastasis of GC cells [15]. Furthermore, circDLST contributed to the metastasis and tumorigenesis of GC by interacting with miR-502-5p [16]. As for circ_0056618, Li et al. uncovered that circ_0056618 could accelerate the malignancy of GC by reducing miR-206 [17]. However, the relationship between circ_0056618 and curcumin is barely known.

miRNAs are short ncRNAs with ∼22 nucleotides and function as important modulators in various types of cancers, including GC [18,19]. It has been reported that miR-194-5p is downregulated in GC, and miR-194-5p overexpression plays a tumor-suppressive role in GC [20,21]. Furthermore, emerging evidence has reported that miRNAs are associated with the anti-tumor properties of curcumin in GC. For example, Sun et al. declared that curcumin repressed the progression of GC by elevating miR-34a [22]. Qiang et al. suggested that curcumin treatment induces GC cell apoptosis and hampers proliferation by modulation of miR-21 [23]. However, whether miR-194-5p is implicated in the anti-tumor effect of curcumin on GC remains unclear.

In this research, we explored the function of curcumin in circ_0056618 and miR-194-5p expression in GC and then elucidated the underlying mechanisms governing the tumor-inhibitory role of curcumin in GC.

## 2 Materials and methods

### 2.1 Sample acquisition

Seventy-one GC tissues and adjacent non-tumor tissues were taken from Qilu Hospital (Qingdao), Cheeloo College of Medicine, Shandong University, and preserved at −80°C prior to use. The patients enrolled in our study did not have other malignant tumors, severe infection, and cognitive impairment history. None of the patients had received any chemotherapy or radiotherapy before this study. The demographic data of 71 patients with GC are shown in Table 1.

**Table 1:** Clinicopathologic features of GC patients

Clinicopathologic features	*n*
Gender	
Male	42
Female	29
Age (years)	
≥60	38
<60	33
Tumor size (cm)	
≥3	43
<3	28
TNM stage	
I–II	21
III–IV	50
Differentiation grade	
Well/moderately	25
Poorly	46
Lymph node metastasis	
Yes	40
No	31

**Informed consent:** Informed consent was obtained from all the individuals included in this study.**Ethical approval:** The research related to human use has complied with all relevant national regulations, institutional policies and is in accordance with the tenets of the Helsinki Declaration, and has been approved by the Ethics Committee of Qilu Hospital (Qingdao), Cheeloo College of Medicine, Shandong University.

### 2.2 Cell culture and curcumin treatment

Normal human gastric epithelial cells (GES-1) and GC cells (HGC-27 and MKN-45) were obtained from Procell (Wuhan, China) and kept in RPMI 1640 medium (Procell) added with 10% fetal bovine serum (FBS; Procell) and 1% penicillin–streptomycin (Procell) at 37°C in a humid incubator containing 5% CO_2_.

Curcumin (Sigma-Aldrich, St. Louis, MO, USA) was dissolved in dimethyl sulfoxide (DMSO; Sigma-Aldrich) at a dose of 50 mM to make stock solution. The stock solution was diluted in the culture medium at a dose of 30 µM. For curcumin treatment, GC cells were subjected to 30 µM curcumin (Sigma-Aldrich) for 24 h in the presence of FBS (Procell). The control groups were subjected to DMSO (Sigma-Aldrich) [23].

### 2.3 Cell transfection

The overexpression vector of circ_0056618 (circ_0056618) and its control (vector), small interfering RNA against circ_0056618 (si-circRNA) and its control (si-NC), and miR-194-5p mimics (miR-194-5p) and its control (miR-NC) were synthesized by GeneCopoeia (Guangzhou, China). HGC-27 and MKN-45 cells were plated into 6-well plates at a density of 1.0 × 10^4^ cells per well and then transfected with the synthetic oligonucleotides (50 nM) or vectors (2 μg) by Lipofectamine 2000 (Invitrogen, Carlsbad, CA, USA), according to the manufacturers’ instructions.

### 2.4 Quantitative reverse transcription polymerase chain reaction (qRT-PCR) assay

The RNA in tissues and cells was extracted using TRIzol reagent (Invitrogen) and digested with DNAse I for 1 h at 37°C to eliminate genomic DNA. The RNAs were then quantified utilizing NanoDrop 2000c spectrophotometer (Thermo Fisher Scientific, Waltham, MA, USA). Then High Capacity cDNA Reverse Transcription Kit (Applied Biosystems, Carlsbad, CA, USA) or TaqMan microRNA Assay kit (Applied Biosystems) was used to reversely transcribe RNAs into cDNAs according to the manufacturers’ instructions. Next, the qRT-PCR was manipulated with SYBR Premix Ex Taq II (Takara, Dalian, China) and specific primers on the StepOnePlus Real-Time PCR System (Applied Biosystems). The primers are presented in Table 2. The expression was calculated using the 2^−ΔΔCt^ method [24]. GAPDH and U6 were used as internal references.

**Table 2:** Primer sequences used for qRT-PCR

Primers	Sequences (5′–3′)	Tm	PCR product	Ct range
circ_0056618-forward	AAGTGGTGATGTCTCGGGAAC	60	209	0.8
circ_0056618-reverse	TTCCCTATCTCCCGCTCCTA	58.8		
miR-194-5p-forward	GCGGCGGTGTAACAGCAACTCC	63.64	98	1.7
miR-194-5p-reverse	ATCCAGTGCAGGGTCCGAGG	63.79		
GAPDH-forward	AAGGTGAAGGTCGGAGTCA	57.86	172	0.8
GAPDH-reverse	GGAAGATGGTGATGGGATTT	55.05		
U6-forward	CGCTTCGGCACATATACTA	54.61	87	1.3
U6-reverse	CGCTTCACGAATTTGCGTGTCA	62.78		

### 2.5 Colony formation assay

GC cells (200 cells/well) were plated into 6-well plates. After adherence and curcumin exposure, the cells were cultivated in normal medium for about 2 weeks. The medium was changed every 3 days. Next, the colonies were fixed in 4% paraformaldehyde (Sangon, Shanghai, China) and dyed with 1% crystal violet (Sangon). The colonies containing >50 cells were counted. This experiment was conducted as previously described [9].

### 2.6 Flow cytometry analysis

For cell cycle analysis, GC cells (1 × 10^4^ cells/well) were sown into 6-well plates. On the next day, the cells were subjected to 30 µM curcumin (Sigma-Aldrich) for 24 h. Next, the cells were harvested and fixed overnight with 70% ethanol. After that, the fixed cells were washed with PBS (Solarbio, Beijing, China) and resuspended in binding buffer at a concentration of 1.0 × 10^6^ cells/mL followed by incubation with propidium iodide (PI; Beyotime, Shanghai, China) for 30 min at 37°C according to manufacturers’ instructions. Finally, the stained cells were analyzed with a FACScan^®^ flow cytometer (Beckman Coulter, Atlanta, GA, USA). For cell apoptosis, the cells (1 × 10^4^ cells/well) were plated into 6-well plates and then administered with 30 µM curcumin (Sigma-Aldrich) for 24 h. After that, the cells were collected, washed, resuspended, and then mixed with 5 μL of Annexin V-fluorescein isothiocyanate (Annexin V-FITC; Beyotime) and 5 μL of PI (Beyotime) for 20 min in the dark according to manufacturers’ instructions. The apoptotic cells were estimated using a FACScan^®^ flow cytometer (Beckman Coulter).

### 2.7 Wound healing assay

GC cells were sowed into 6-well plates (2 × 10^3^ cells/well) and grown until 100% confluence. Then the cells were scratched utilizing a pipette tip and washed with PBS (Solarbio) to remove the detached cells followed by treatment with curcumin for 24 h (Sigma-Aldrich). The scratched areas were photographed at 0 and 48 h at a magnification of 40× and the wound closure was recorded to assess cell migration capacity. The experiment was conducted as previously reported [25].

### 2.8 Transwell assay

The 24-well transwell inserts (Corning Incorporated, Corning, NY, USA) coated with or without Matrigel (Solarbio) were adopted to evaluate cell invasion and migration, respectively. Briefly, after indicated transfection and treatment, HGC-27 and MKN-45 cells (2 × 10^4^ cells/well) were suspended into serum-free medium and then added into the top compartment of chambers. The bottom compartment of chambers was filled with 500 μL of culture medium to act as a chemoattractive. After 24 h, the transferred cells were dyed with 1% crystal violet (Sangon) and determined under an inverted microscope (Olympus, Tokyo, Japan) at a magnification of 100×.

### 2.9 Dual-luciferase reporter assay

The sequences of wild-type (WT) circ_0056618 containing the putative miR-194-5p binding sites were cloned into pmirGLO plasmid (Promega, Fitchburg, WI, USA) to establish the luciferase reporter vector circ_0056618 WT. Similarly, the sequences of mutant (MUT) circ_0056618 lacking the miR-194-5p-binding sites were inserted into pmirGLO plasmid (Promega) to generate circ_0056618 MUT. GC cells were seeded into 6-well plates (5 × 10^4^ cells/well) and then co-transfected with circ_0056618 WT or circ_0056618 MUT (100 ng) and miR-194-5p or miR-NC(50 nM). After 48 h, the Renilla and firefly luciferase activities were measured with a Dual-Luciferase Reporter Assay System (Promega).

### 2.10 RNA pull-down assay

RNA pull-down assay was executed utilizing the Pierce Magnetic RNA-Protein Pull-Down Kit (Thermo Fisher Scientific). In brief, biotin-labeled wild-type miR-194-5p (Bio-miR-194-5p-WT), mutant miR-194-5p (Bio-miR-194-5p-MUT), or Bio-miR-NC was transfected into GC cells (1 × 10^4^ cells/well) and cultivated for 24 h. Afterward, the cells were collected, lysed, and incubated with streptavidin-coated magnetic beads. Biotin-coupled RNA complexes were pulled down, bound RNAs were extracted, and then the enrichment of circ_0056618 was estimated by qRT-PCR analysis.

### 2.11 Western blot assay

Total protein in GC cells was extracted utilizing RIPA buffer (Beyotime) and determined utilizing a BCA protein assay kit (Tiangen, Beijing, China). Then the equal amount of protein (30 μg) was separated by sodium dodecyl sulfonate–polyacrylamide gel (Solarbio) and blotted onto polyvinylidenedifluoride membranes (Amersham Biosciences, Chicago, IL, USA). After blocking in 5% defatted milk for 1 h, the membranes were cultivated overnight with primary antibodies against CyclinD1 (1:2,000; bs-0623R; Bioss, Beijing, China), E-cadherin (1:2,000; bs-10009R; Bioss), N-cadherin (1:2,000; bs-1172R; Bioss), or GAPDH (1:5,000; bs-2188R; Bioss) at 4°C followed by interaction with HRP-conjugated secondary antibody (1:5,000; bs-0295M-HRP; Bioss) for 1.5 h at room temperature. The protein bands were visualized with an ECL reagent (Vazyme, Nanjing, China) and analyzed via ImageJ v1.8.0 (National Institutes of Health).

### 2.12 Murine xenograft model

The BALB/c nude mice (4–6 weeks old) were obtained from Vital River Laboratory (Beijing, China) and divided into four groups (*n* = 6/group). For control and curcumin groups, 2 × 10^6^ MKN-45 cells suspended in PBS (Solarbio) were subcutaneously implanted into the flank of the nude mice, and DMSO (Sigma-Aldrich) or 30 µM curcumin (Sigma-Aldrich) was intraperitoneally administered into the mice every 7 days. For curcumin + vector and curcumin + circ_0056618 groups, circ_0056618 or vector transfected MKN-45 cells were implanted into the nude mice and then 30 µM curcumin (Sigma-Aldrich) was intraperitoneally administered into the mice every 7 days. Tumor volume was examined every 7 days and computed via the equation: (length × width^2^)/2. The mice were euthanized after 28 days through cervical dislocation and the neoplasms were weighted and preserved for qRT-PCR assay.

**Ethical approval:** The research related to animal use has been complied with all the relevant national regulations and institutional policies for the care and use of animals and was approved by the Ethics Committee of Animal Research of Qilu Hospital (Qingdao), Cheeloo College of Medicine, Shandong University.

### 2.13 Statistical analysis

Each experiment was conducted in triplicate. The data were analyzed with GraphPad Prism 7 and exhibited as mean ± standard deviation. The differences in two groups and three groups were estimated using Student’s *t*-test and one-way analysis of variance followed by Tukey’s test, respectively. Pearson’s correlation coefficient analysis estimated the linear correlation between circ_0056618 and miR-194-5p in GC tissues after Agostino–Pearson, Kolmogorov–Smirnov, and Shapiro–Wilk methods were used for the analysis of normality distribution of the data. Differences were considered as significant where *P*-value < 0.05 is represented as * and *P*-value < 0.01 is represented as **.

## 3 Results

### 3.1 circ_0056618 was upregulated and miR-194-5p was downregulated in GC tissues

**Figure 1:**
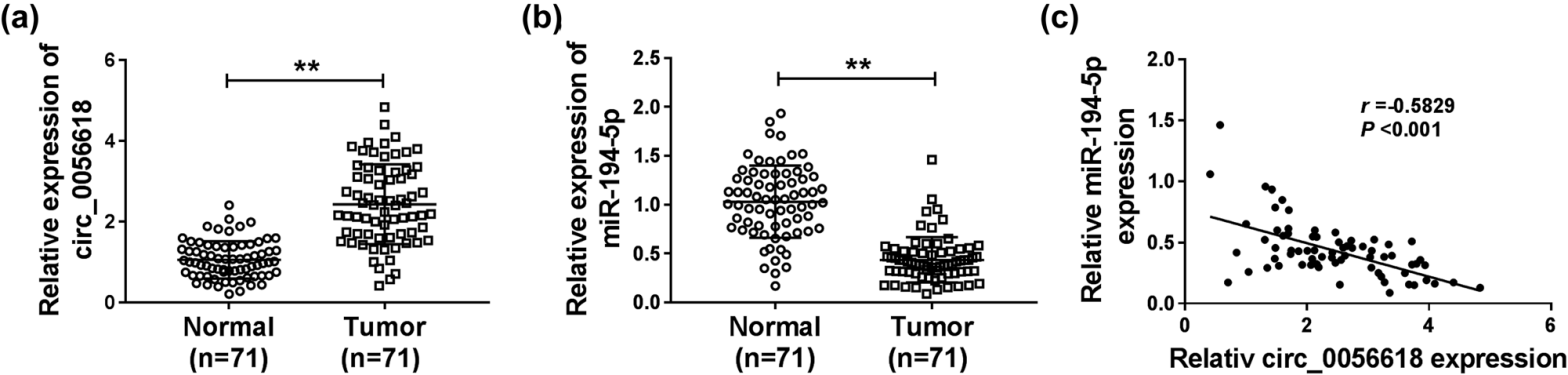
High expression of circ_0056618 and low expression of miR-194-5p in GC tissues. (a and b) qRT-PCR analysis was conducted for circ_0056618 and miR-194-5p expression levels in GC tissues and adjacent normal tissues. (c) The correlation between the levels of circ_0056618 and miR-194-5p in GC tissues was analyzed by Pearson’s correlation coefficient analysis. **P < 0.01.

Initially, qRT-PCR assay was conducted to test the expression levels of circ_0056618 and miR-194-5p in GC tissues and adjacent non-tumor tissues. The results showed that circ_0056618 was highly expressed and miR-194-5p was lowly expressed in GC tissues compared to non-tumor tissues (Figure 1a and b). Pearson’s correlation coefficient analysis estimated that miR-194-5p level was negatively correlated with circ_0056618 level in GC tissues (Figure 1c). These results indicated that circ_0056618 and miR-194-5p might be related to GC progression.

### 3.2 circ_0056618 overexpression ameliorated the effects of curcumin treatment on GC cell colony formation, cell cycle process, and apoptosis

**Figure 2:**
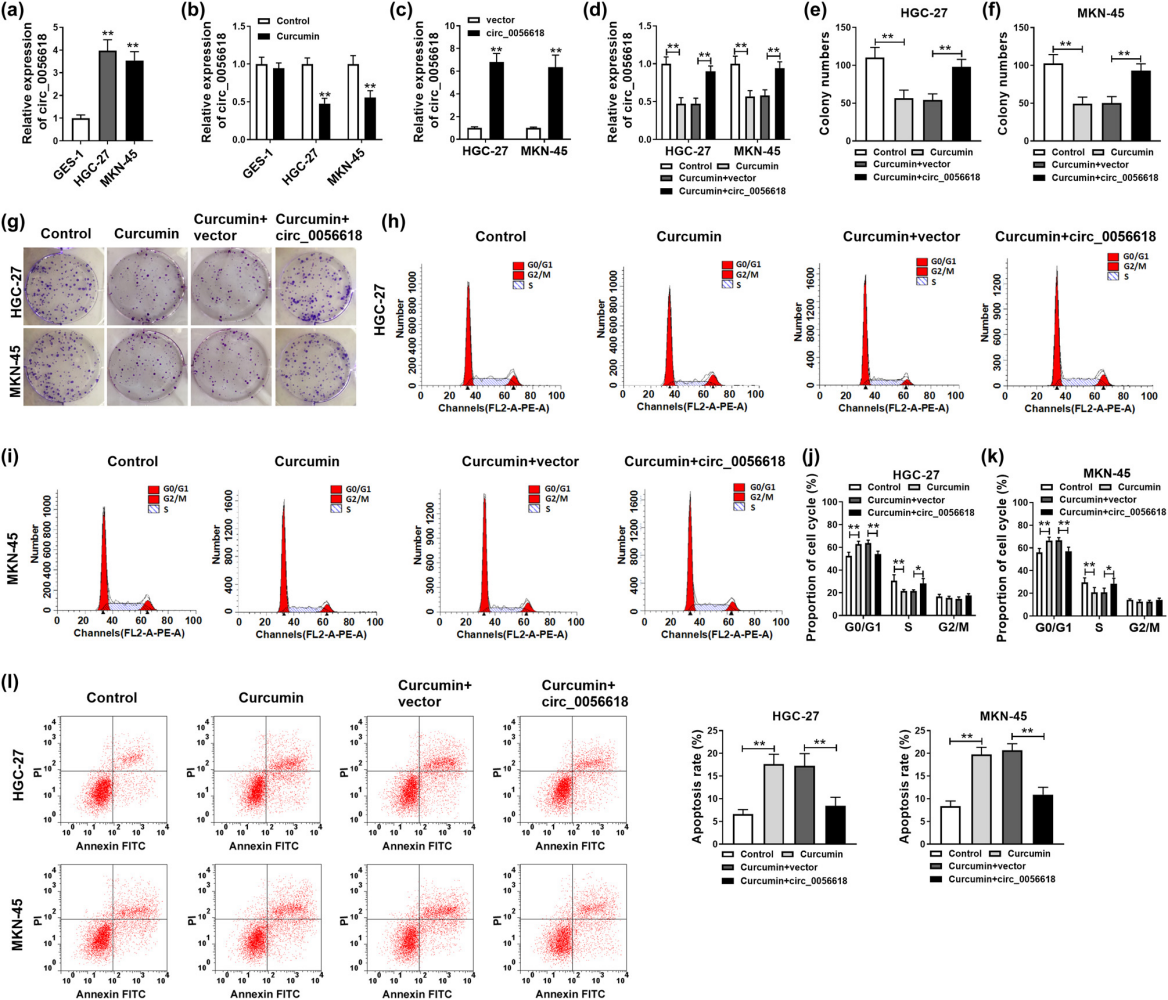
Curcumin relieved the malignant cell phenotypes of GC cells by downregulating circ_0056618. (a) qRT-PCR assay was conducted for circ_0056618 level in GES-1, HGC-27, and MKN-45 cells. (b) The expression level of circ_0056618 in curcumin-treated GES-1, HGC-27, and MKN-45 cells was determined by qRT-PCR assay. (c) The expression of circ_0056618 in HGC-27 and MKN-45 cells transfected with circ_0056618 or vector was examined by qRT-PCR assay. (d–l) HGC-27 and MKN-45 cells were assigned to four groups: control, curcumin, curcumin + vector, and curcumin + circ_0056618. (d) The expression of circ_0056618 in HGC-27 and MKN-45 cells was detected by qRT-PCR assay. (f–g) The colony formation ability of HGC-27 and MKN-45 cells was examined by colony formation assay. (h–l) The cell cycle process and apoptosis of HGC-27 and MKN-45 cells were analyzed through flow cytometry analysis. **P < 0.01, *P < 0.05.

As shown in Figure 2a, circ_0056618 level was elevated in GC cell lines (HGC-27 and MKN-45) compared to that in GES-1 cell line. Then HGC-27 and MKN-45 cells were subjected to 30 µM curcumin for 24 h followed by qRT-PCR assay for circ_0056618 expression level. As a result, curcumin treatment led to an inhibition in circ_0056618 level in HGC-27 and MKN-45 cells compared to control groups, but curcumin did not affect the expression of circ_0056618 in GES-1 cells (Figure 2b). Next, the transfection of circ_0056618 increased the level of circ_0056618 in HGC-27 and MKN-45 cells, suggesting the successful transfection of circ_0056618 (Figure 2c). To explore the association between curcumin and circ_0056618 in the regulation of GC progression, HGC-27 and MKN-45 cells were treated with control, curcumin, curcumin + vector, or curcumin + circ_0056618. As shown in Figure 2d, curcumin-mediated downregulation on circ_0056618 expression was reversed by circ_0056618 overexpression vector transfection. The results of colony formation assay indicated that the colony formation ability of HGC-27 and MKN-45 cells was suppressed following curcumin exposure, while circ_0056618 overexpression overturned the effect (Figure 2e–g). Flow cytometry analysis showed that the proportion of HGC-27 and MKN-45 cells was increased in the G0/G1 phase and reduced in the S phase after curcumin treatment, whereas the impacts were reversed by elevating circ_0056618 (Figure 2h–k). In addition, flow cytometry analysis also exhibited that curcumin treatment facilitated the apoptosis of HGC-27 and MKN-45 cells, while circ_0056618 overexpression abated this effect (Figure 2l). Collectively, circ_0056618 overexpression weakened the effects of curcumin on the malignant behaviors of GC cells.

### 3.3 Overexpression of circ_0056618 reversed the inhibitory effects of curcumin on GC cell migration and invasion

**Figure 3:**
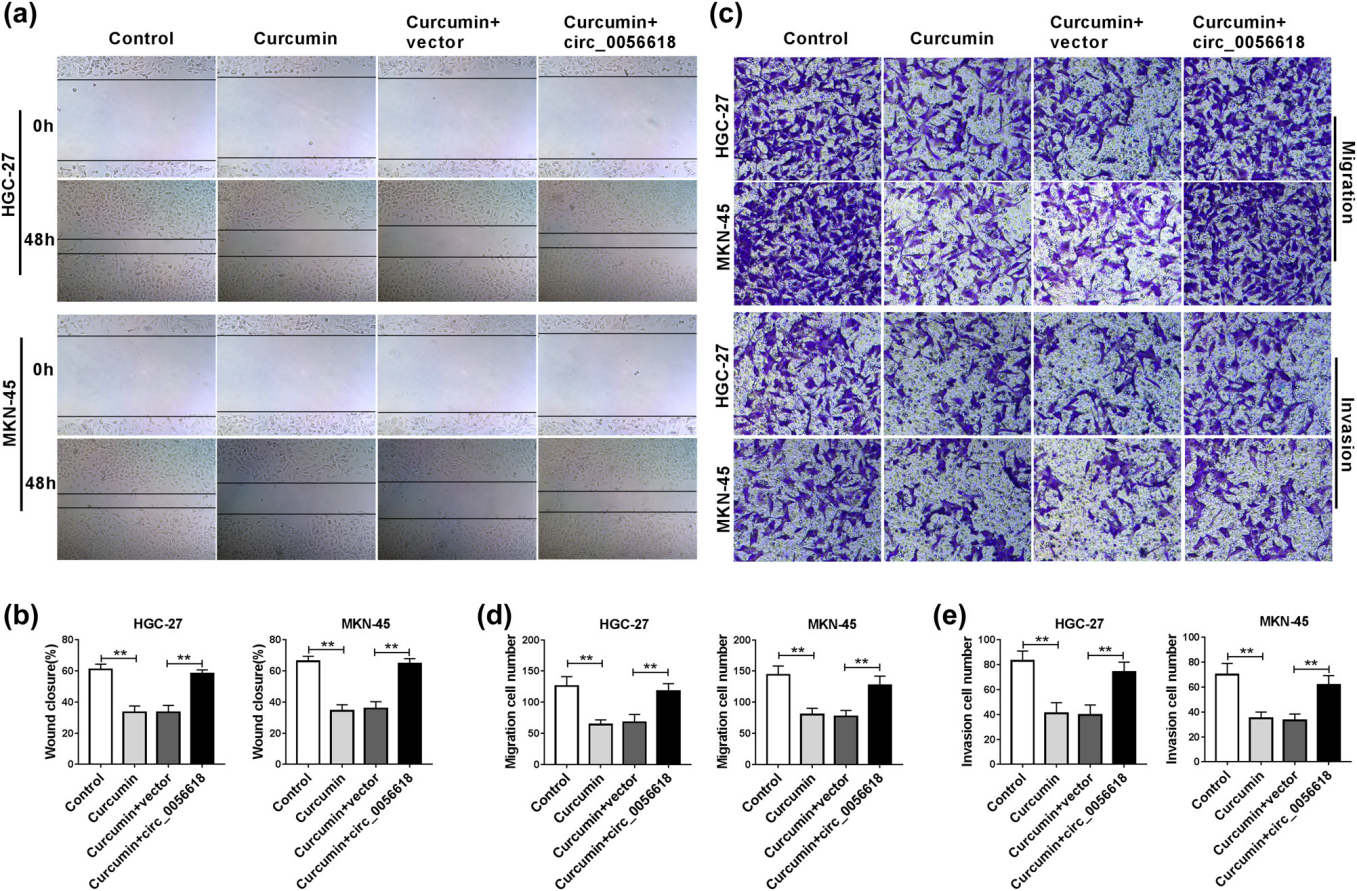
circ_0056618 overexpression reversed the effects of curcumin on cell migration and invasion in GC cells. HGC-27 and MKN-45 cells were assigned to four groups: control, curcumin, curcumin + vector, and curcumin + circ_0056618. (a and b) The migration of HGC-27 and MKN-45 cells was evaluated by wound healing assay. (c–e) The migration and invasion of HGC-27 and MKN-45 cells were assessed via transwell assay. **P < 0.01.

To investigate whether curcumin affected the migration and invasion of GC cells through regulating circ_0056618, wound healing assay and transwell assay were carried out. Wound healing assay exhibited that curcumin treatment suppressed the ability of wound closure, indicating that the migration ability of HGC-27 and MKN-45 cells was suppressed, which was rescued by increasing circ_0056618 (Figure 3a and b). Moreover, transwell assay showed that the migration and invasion capacities of HGC-27 and MKN-45 cells were repressed by curcumin exposure, while circ_0056618 overexpression abrogated the effects (Figure 3c–e). Taken together, curcumin restrained GC cell migration and invasion by modulation of circ_0056618.

### 3.4 circ_0056618 functioned as a sponge of miR-194-5p

**Figure 4:**
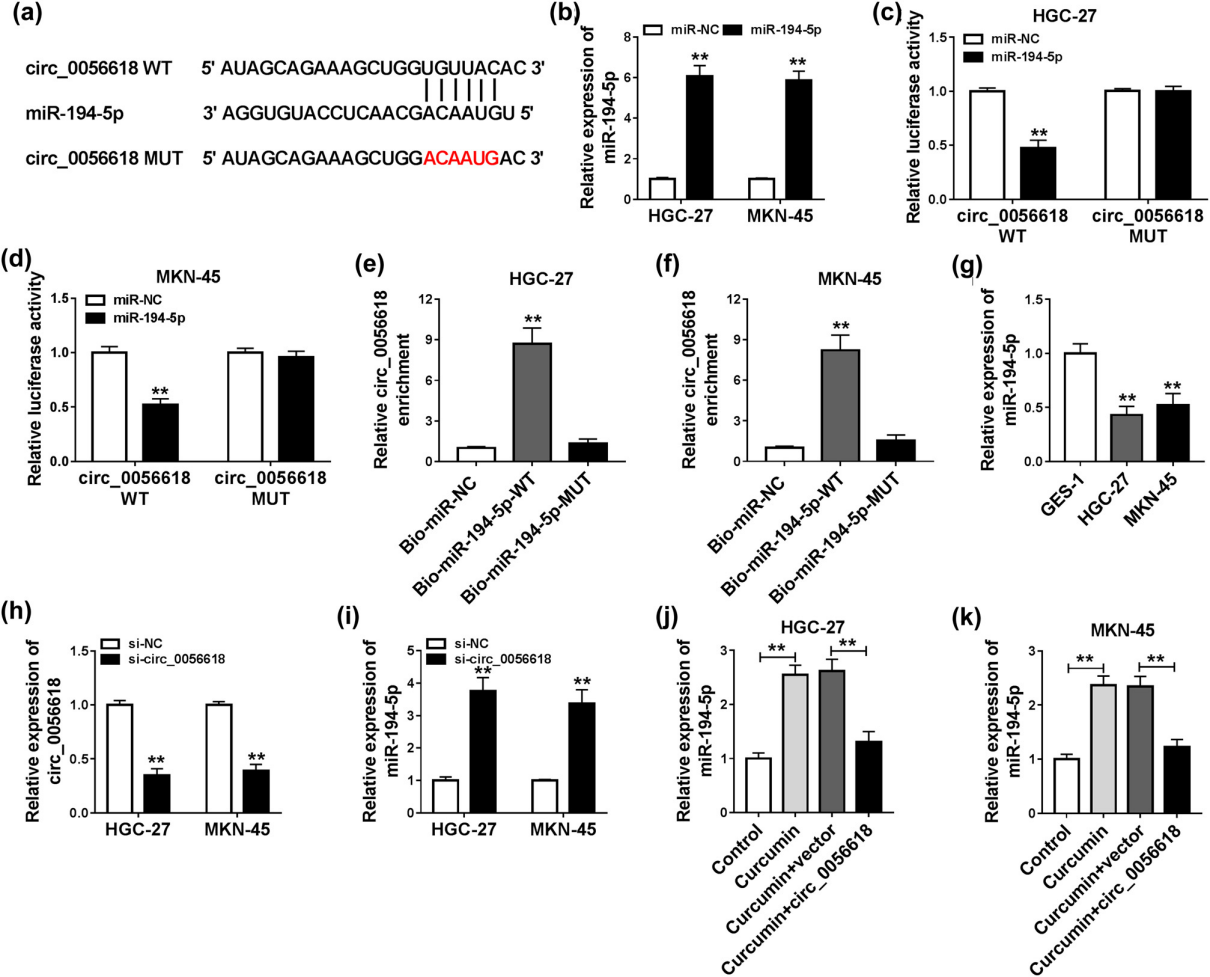
miR-194-5p was a target of circ_0056618. (a) The potential binding sites between miR-194-5p and circ_0056618 are shown. (b) The expression level of miR-194-5p in HGC-27 and MKN-45 cells transfected with miR-NC or miR-194-5p was determined by qRT-PCR assay. (c and d) The luciferase activity in miR-194-5p/miR-NC and circ_0056618 WT/circ_0056618 MUT co-transfected HGC-27 and MKN-45 cells was measured by dual-luciferase reporter assay. (e and f) After RNA pull-down assay, the enrichment of circ_0056618 in HGC-27 and MKN-45 cell lysates was determined by qRT-PCR assay. (g) qRT-PCR assay was conducted for miR-194-5p expression in GES-1, HGC-27, and MKN-45 cells. (h and i) The expression levels of circ_0056618 and miR-194-5p in HGC-27 and MKN-45 cells transfected with si-NC or si-circRNA were examined through qRT-PCR assay. (j and k) After HGC-27 and MKN-45 cells were treated with control, curcumin, curcumin + vector, or curcumin + circ_0056618, the expression level of miR-194-5p was detected through qRT-PCR assay. **P < 0.01.

Through analyzing online tool Circinteractome (https://circinteractome.nia.nih.gov), we found that miR-194-5p contained the potential binding sites of circ_0056618, indicating that miR-194-5p might be a target of circ_0056618 (Figure 4a). As exhibited in Figure 4b, miR-194-5p transfection increased the level of miR-194-5p in HGC-27 and MKN-45 cells compared to miR-NC control groups. Then dual-luciferase reporter assay and RNA pull-down assay were performed to verify the interaction between circ_0056618 and miR-194-5p. Dual-luciferase reporter assay presented that miR-194-5p transfection inhibited the luciferase activity of circ_0056618 WT, but had no effect on the luciferase activity of circ_0056618 MUT in both HGC-27 and MKN-45 cells (Figure 4c and d). RNA pull-down assay indicated that circ_0056618 could be abundantly enriched by Bio-miR-194-5p-WT probe in both HGC-27 and MKN-45 cells compared to Bio-miR-NC or Bio-miR-194-5p-MUT groups (Figure 4e and f). As expected, there was a lower expression of miR-194-5p in HGC-27 and MKN-45 cells than in GES-1 cells (Figure 4g). Moreover, we found that si-circRNA transfection led to a decrease in circ_0056618 expression and an increase in miR-194-5p expression in both HGC-27 and MKN-45 cells in comparison with control groups (Figure 4h and i). Additionally, our results exhibited that curcumin treatment increased miR-194-5p level in HGC-27 and MKN-45 cells, whereas circ_0056618 overexpression restored this effect (Figure 4j and k). All these results suggested that circ_0056618 directly interacted with miR-194-5p to negatively regulate miR-194-5p expression in GC cells.

### 3.5 miR-194-5p overexpression reversed the promotional effects of circ_0056618 on the malignant behaviors of curcumin-treated GC cells

**Figure 5:**
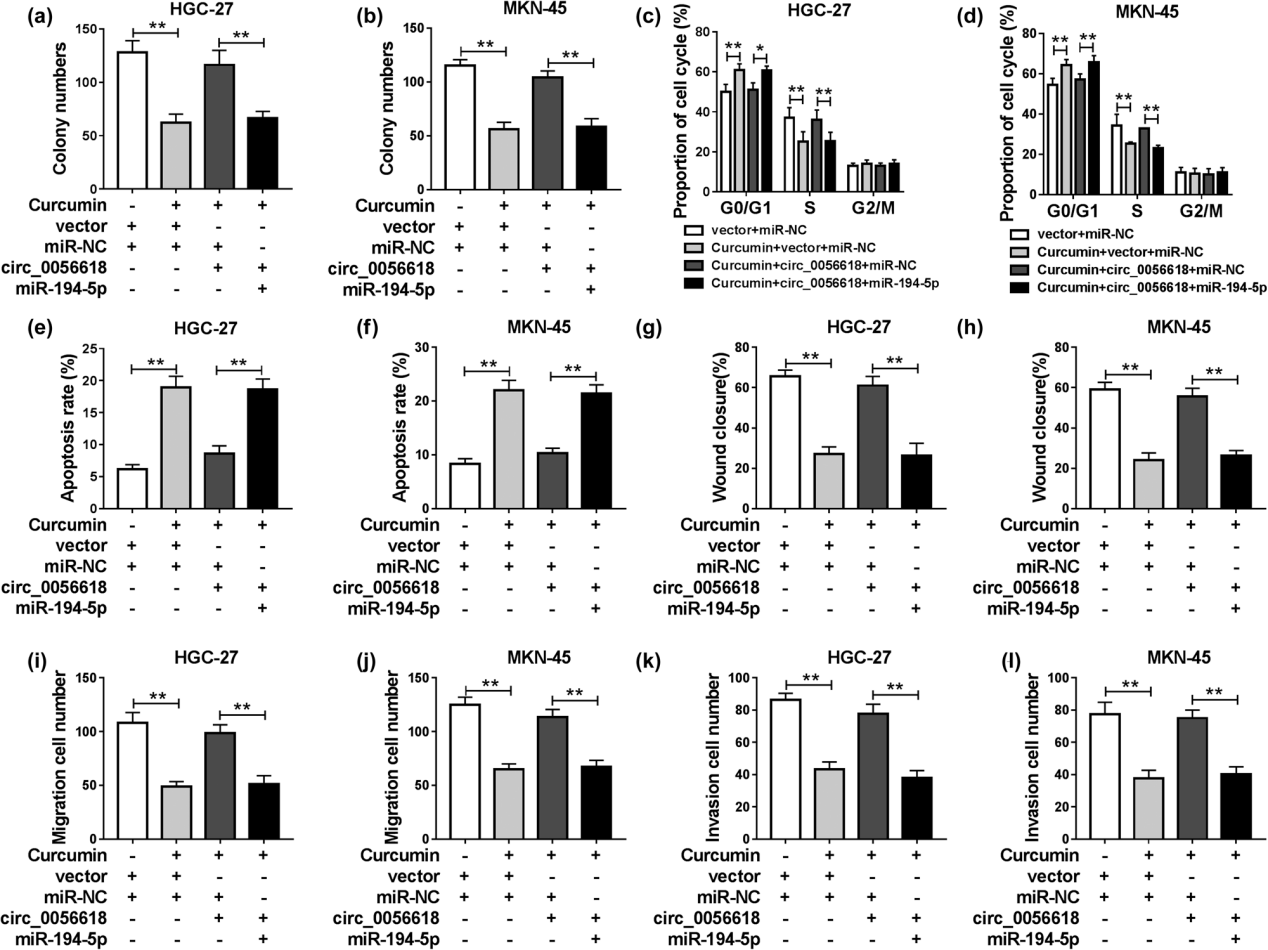
Curcumin relieved the malignant behaviors of GC cells by regulating circ_0056618/miR-194-5p axis. HGC-27 and MKN-45 cells were administered with vector + miR-NC, curcumin + vector + miR-NC, curcumin + circ_0056618 + miR-NC, or curcumin + circ_0056618 + miR-194-5p. (a and b) The colony formation of HGC-27 and MKN-45 cells was assessed by colony formation assay. (c–f) Flow cytometry analysis was conducted for cell cycle process and apoptosis. (g–l) The migration and invasion abilities of HGC-27 and MKN-45 cells were evaluated by wound healing assay and transwell assay. **P < 0.01.

To further explore whether curcumin could decelerate the progression of GC cells through circ_0056618/miR-194-5p axis, HGC-27 and MKN-45 cells were treated with vector + miR-NC, curcumin + vector + miR-NC, curcumin + circ_0056618 + miR-NC, or curcumin + circ_0056618 + miR-194-5p. As demonstrated by colony formation, the promotional effect of circ_0056618 on cell viability could be reversed by increasing miR-194-5p in curcumin-treated HGC-27 and MKN-45 cells in comparison with control groups (Figure 5a and b). Flow cytometry analysis indicated that the promotional role in cell cycle process and the suppressive role in cell apoptosis in curcumin-treated HGC-27 and MKN-45 cells mediated by circ_0056618 overexpression were all abolished by the elevation of miR-194-5p (Figure 5c–f). In addition, the results of wound healing assay and transwell assay demonstrated that the promotional effects of circ_0056618 on cell migration and invasion in curcumin-treated HGC-27 and MKN-45 cells were rescued by elevating miR-194-5p (Figure 5g–l). To sum up, circ_0056618 could promote cell progression in curcumin-treated GC cells by targeting miR-194-5p.

### 3.6 Effects of curcumin on the levels of CyclinD1, E-cadherin, and N-cadherin in GC cells

**Figure 6:**
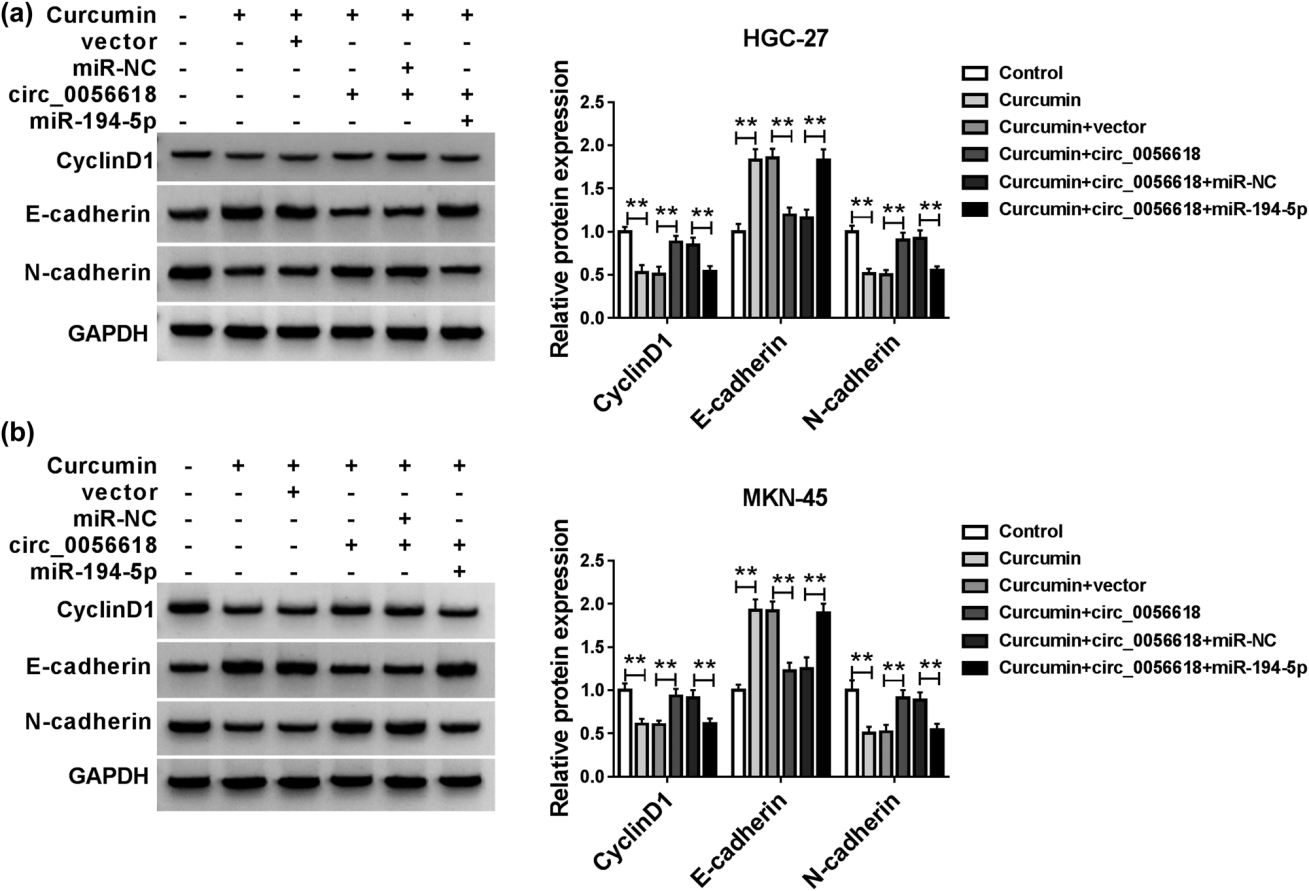
Curcumin altered the expression of CyclinD1, E-cadherin, and N-cadherin in GC cells by modulation of circ_0056618/miR-194-5p axis. (a and b) After HGC-27 and MKN-45 cells were treated with control, curcumin, curcumin + vector, curcumin + circ_0056618, curcumin + circ_0056618 + miR-NC, or curcumin + circ_0056618 + miR-194-5p, the protein levels of CyclinD1, E-cadherin, and N-cadherin were measured by western blot assay. **P < 0.01.

Subsequently, we determined the effects of curcumin on the expression levels of CyclinD1 and EMT-associated markers (E-cadherin and N-cadherin) in HGC-27 and MKN-45 cells by western blot assay. Our results showed that curcumin treatment reduced the protein levels of CyclinD1 and N-cadherin and enhanced the protein level of E-cadherin in HGC-27 and MKN-45 cells, while circ_0056618 overexpression abrogated the impacts; moreover, the elevation of miR-194-5p further overturned the effects of circ_0056618 overexpression on CyclinD1, E-cadherin, and N-cadherin levels (Figure 6a and b). These findings indicated that curcumin could modulate the expression of CyclinD1, E-cadherin, and N-cadherin in GC cells through circ_0056618/miR-194-5p axis.

### 3.7 Curcumin blocked the tumorigenesis of GC *in vivo* by regulating circ_0056618

**Figure 7:**
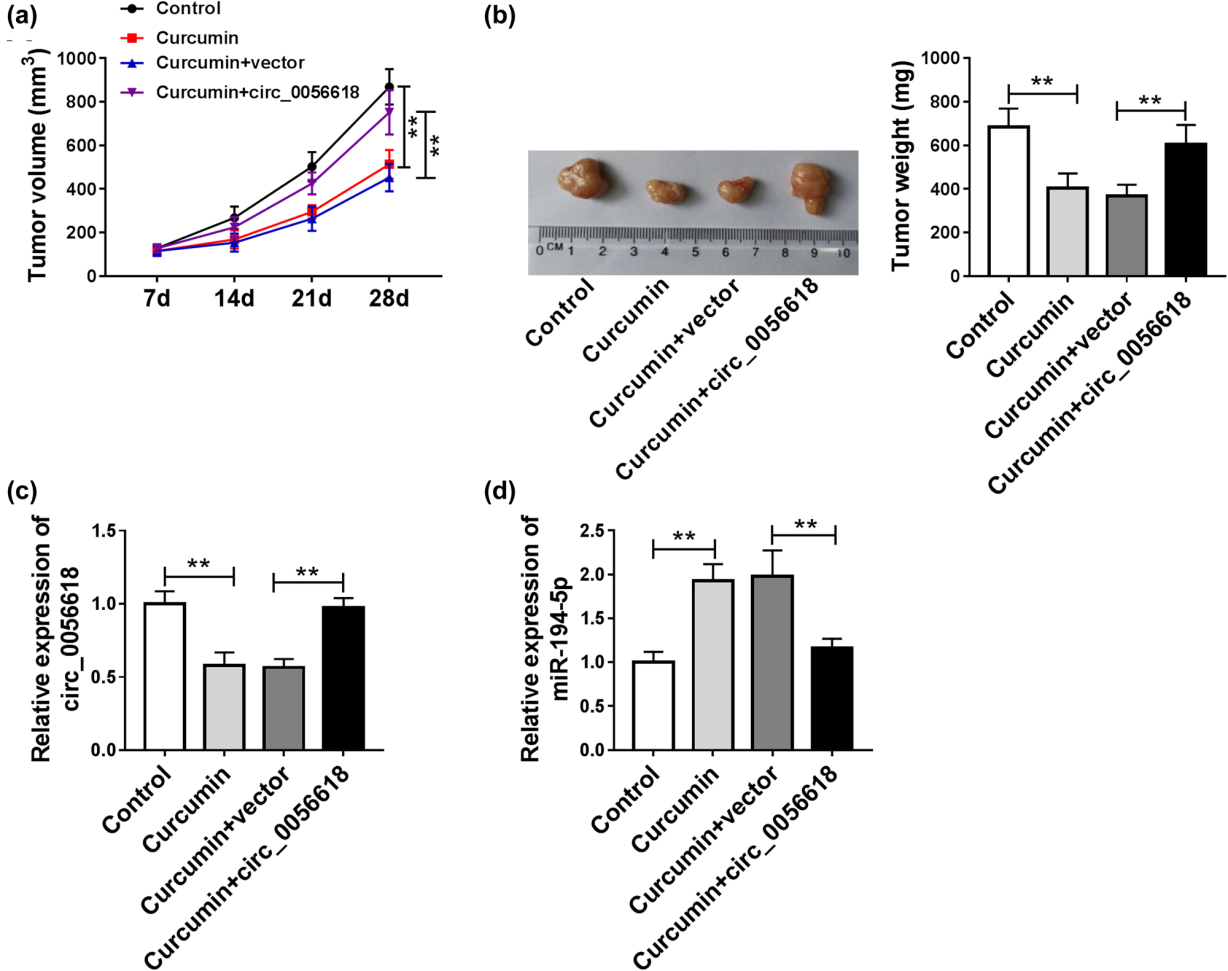
Curcumin hampered tumor growth *in vivo*. (a) Tumor volume was examined every 7 days. (b) Tumor weight was examined on day 28. (c and d) The expression levels of circ_0056618 and miR-194-5p in the collected tumor tissues were measured by qRT-PCR assay. **P < 0.01.

At last, the function of curcumin in GC growth *in vivo* was determined through constructing the murine xenograft model. As shown in Figure 7a and b, tumor size and weight were restrained in curcumin groups compared to control groups, and tumor size and weight were increased in curcumin + circ_0056618 groups compared to curcumin + vector groups. Moreover, we found that circ_0056618 level was decreased and miR-194-5p level was increased in the tumor tissues collected from curcumin groups, while the effects were rescued in curcumin + circ_0056618 groups (Figure 7c and d). These results suggested that curcumin hampered tumor growth of GC *in vivo* by regulating circ_0056618.

## 4 Discussion

A growing body of evidence has demonstrated that curcumin restrains the malignant biological behaviors of GC cells through different pathways [26–28]. Herein, we discovered that curcumin was able to inhibit GC cell growth and motility and induce apoptosis by suppressing circ_0056618 and elevating miR-194-5p.

Previous research have verified that curcumin exerts the anti-tumor effect mainly by repressing tumor cell proliferation and motility and facilitating apoptosis. For example, curcumin treatment restrained Rb cell proliferation, invasion, and migration and accelerated apoptosis [9]. Curcumin suppressed the growth and cell cycle process and facilitated the apoptosis of GC cells [22]. In line with these reports, we demonstrated that curcumin treatment restrained cell colony formation, migration, and invasion and accelerated cell cycle arrest and apoptosis in GC cells *in vitro* and blocked tumorigenesis of GC *in vivo*, indicating that curcumin might be a candidate agent for GC therapy.

Zheng et al. suggested that circ_0056618 level was enhanced in colorectal cancer (CRC) and promoted CRC cell growth, angiogenesis, and migration by reducing miR-206 and elevating CXCR4 and VEGF-A [29]. Li et al. unraveled that circ_0056618 was elevated in GC and facilitated cell proliferation and motility in GC [17]. In this study, we also observed that there was an increase in circ_0056618 level in GC tissues and cells. Moreover, we found that curcumin treatment predominantly decreased the expression of circ_0056618 in GC cells. Thus, we further explored whether circ_0056618 could participate in regulating curcumin-mediated malignant phenotypes of GC cells. As a result, overexpression of circ_0056618 ameliorated curcumin-mediated anti-proliferation, anti-metastasis, and pro-apoptosis effects on GC cells, suggesting that curcumin could decelerate GC development by reducing circ_0056618 expression.

Subsequently, the underlying mechanism of how curcumin regulates GC progression was further investigated. miR-194-5p has been demonstrated to be the target of several circRNAs, such as circ_0023028 [30], circ-USP1 [31], and circ_0001971 [32]. Through bioinformatics analysis, dual-luciferase reporter assay and RNA pull-down assay, miR-194-5p was verified to be the target of circ_0056618 for the first time. Our results showed that miR-194-5p was weakly expressed in GC tissues and cells, and circ_0056618 could negatively regulate miR-194-5p expression in GC cells and curcumin-treated GC cells. Previous studies showed that miR-194-5p had the capacity to repress GC cell growth and metastasis [33,34]. In this study, our results exhibited that the elevation of miR-194-5p effectively reversed the impacts of circ_0056618 on cell growth, apoptosis, and metastasis in curcumin-treated GC cells, indicating that circ_0056618 promoted curcumin-mediated GC development by interacting with miR-194-5p.

In summary, curcumin treatment could repress GC cell growth and metastasis and promote apoptosis partly by regulation of circ_0056618/miR-194-5p axis. The findings facilitated our understanding on the mechanism of curcumin in GC therapy and indicated that curcumin might be a potential therapeutic drug for GC. In addition, accumulating evidence showed that curcumin might prevent GC through regulation of oncogenic pathways [35]. However, the underlying mechanisms still need further investigation.

## References

[1] Lordick F, Janjigian YY. Clinical impact of tumour biology in the management of gastroesophageal cancer. Nat Rev Clin Oncol. 2016;13(6):348–60. 10.1038/nrclinonc.2016.15 PMC5521012 26925958

[2] de Martel C, Forman D, Plummer M. Gastric cancer: epidemiology and risk factors. Gastroenterol Clin North Am. 2013;42(2):219–40. 10.1016/j.gtc.2013.01.003 23639638

[3] Kanda M, Kodera Y. Recent advances in the molecular diagnostics of gastric cancer. World J Gastroenterol. 2015;21(34):9838–52. 10.3748/wjg.v21.i34.9838 PMC4566379 26379391

[4] Shen L, Shan YS, Hu HM, Price TJ, Sirohi B, Yeh KH, et al. Management of gastric cancer in Asia: resource-stratified guidelines. Lancet Oncol. 2013;14(12):e535–47. 10.1016/S1470-2045(13)70436-4 24176572

[5] Jemal A, Siegel R, Ward E, Hao Y, Xu J, Thun MJ. Cancer statistics, 2009. CA Cancer J Clin. 2009;59(4):225–49. 10.3322/caac.20006 19474385

[6] Shanmugam MK, Rane G, Kanchi MM, Arfuso F, Chinnathambi A, Zayed ME, et al. The multifaceted role of curcumin in cancer prevention and treatment. Molecules. 2015;20(2):2728–69. 10.3390/molecules20022728 PMC6272781 25665066

[7] Lopez-Lazaro M. Anticancer and carcinogenic properties of curcumin: considerations for its clinical development as a cancer chemopreventive and chemotherapeutic agent. Mol Nutr Food Res. 2008;52(Suppl 1):S103–27. 10.1002/mnfr.200700238 18496811

[8] Wang Q, Qu C, Xie F, Chen L, Liu L, Liang X, et al. Curcumin suppresses epithelial-to-mesenchymal transition and metastasis of pancreatic cancer cells by inhibiting cancer-associated fibroblasts. Am J Cancer Res. 2017;7(1):125–33. PMC5250686 28123853

[9] Li Y, Sun W, Han N, Zou Y, Yin D. Curcumin inhibits proliferation, migration, invasion and promotes apoptosis of retinoblastoma cell lines through modulation of miR-99a and JAK/STAT pathway. BMC Cancer. 2018;18(1):1230. 10.1186/s12885-018-5130-y PMC6288931 30526546

[10] Sun Y, Liu L, Wang Y, He A, Hu H, Zhang J, et al. Curcumin inhibits the proliferation and invasion of MG-63 cells through inactivation of the p-JAK2/p-STAT3 pathway. Onco Targets Ther. 2019;12:2011–21. 10.2147/OTT.S172909 PMC6421868 30936718

[11] Wang K, Tan SL, Lu Q, Xu R, Cao J, Wu SQ, et al. Curcumin suppresses microRNA-7641-mediated regulation of p16 expression in bladder cancer. Am J Chin Med. 2018;46(6):1357–68. 10.1142/S0192415X18500714 30149755

[12] Gu X, Zhang Q, Zhang W, Zhu L. Curcumin inhibits liver metastasis of gastric cancer through reducing circulating tumor cells. Aging (Albany NY). 2019;11(5):1501–9. 10.18632/aging.101848 PMC6428112 30844765

[13] Silva G, Teixeira Lima F, Seba V, Mendes Lourenco AL, Lucas TG, de Andrade BV, et al. Curcumin analog CH-5 suppresses the proliferation, migration, and invasion of the human gastric cancer cell line HGC-27. Molecules. 2018;23(2):279. 10.3390/molecules23020279 PMC6017500 29385675

[14] Zhou R, Wu Y, Wang W, Su W, Liu Y, Wang Y, et al. Circular RNAs (circRNAs) in cancer. Cancer Lett. 2018;425:134–42. 10.1016/j.canlet.2018.03.035 29625140

[15] Li J, Zhen L, Zhang Y, Zhao L, Liu H, Cai D, et al. circ-104916 is downregulated in gastric cancer and suppresses migration and invasion of gastric cancer cells. Onco Targets Ther. 2017;10:3521–9. 10.2147/OTT.S136347 PMC5522828 28761361

[16] Zhang J, Hou L, Liang R, Chen X, Zhang R, Chen W, et al. circDLST promotes the tumorigenesis and metastasis of gastric cancer by sponging miR-502-5p and activating the NRAS/MEK1/ERK1/2 signaling. Mol Cancer. 2019;18(1):80. 10.1186/s12943-019-1015-1 PMC6449953 30953514

[17] Li H, Yao G, Feng B, Lu X, Fan Y. circ_0056618 and CXCR4 act as competing endogenous in gastric cancer by regulating miR-206. J Cell Biochem. 2018;119(11):9543–51. 10.1002/jcb.27271 30129184

[18] van Kouwenhove M, Kedde M, Agami R. MicroRNA regulation by RNA-binding proteins and its implications for cancer. Nat Rev Cancer. 2011;11(9):644–56. 10.1038/nrc3107 21822212

[19] Wang QX, Zhu YQ, Zhang H, Xiao J. Altered miRNA expression in gastric cancer: a systematic review and meta-analysis. Cell Physiol Biochem. 2015;35(3):933–44. 10.1159/000369750 25633747

[20] Qu F, Cao P. Long noncoding RNA SOX2OT contributes to gastric cancer progression by sponging miR-194-5p from AKT2. Exp Cell Res. 2018;369(2):187–96. 10.1016/j.yexcr.2018.05.017 29782828

[21] Ding Z, Lan H, Xu R, Zhou X, Pan Y. LncRNA TP73-AS1 accelerates tumor progression in gastric cancer through regulating miR-194-5p/SDAD1 axis. Pathol Res Pract. 2018;214(12):1993–9. 10.1016/j.prp.2018.09.006 30279010

[22] Sun C, Zhang S, Liu C, Liu X. Curcumin promoted miR-34a expression and suppressed proliferation of gastric cancer cells. Cancer Biother Radiopharm. 2019;34(10):634–41. 10.1089/cbr.2019.2874 31539270

[23] Qiang Z, Meng L, Yi C, Yu L, Chen W, Sha W. Curcumin regulates the miR-21/PTEN/Akt pathway and acts in synergy with PD98059 to induce apoptosis of human gastric cancer MGC-803 cells. J Int Med Res. 2019;47(3):1288–97. 10.1177/0300060518822213 PMC6421392 30727807

[24] Livak KJ, Schmittgen TD. Analysis of relative gene expression data using real-time quantitative PCR and the 2(-Delta Delta C(T)) Method. Methods. 2001;25(4):402–8. 10.1006/meth.2001.1262 11846609

[25] Li Y, Jiang B, He Z, Zhu H, He R, Fan S, et al. circIQCH sponges miR-145 to promote breast cancer progression by upregulating DNMT3A expression. Aging (Albany NY). 2020;12(15):15532–45. 10.18632/aging.103746 PMC7467367 32756009

[26] Cai XZ, Wang J, Li XD, Wang GL, Liu FN, Cheng MS, et al. Curcumin suppresses proliferation and invasion in human gastric cancer cells by downregulation of PAK1 activity and cyclin D1 expression. Cancer Biol Ther. 2009;8(14):1360–8. 10.4161/cbt.8.14.8720 19448398

[27] Yu LL, Wu JG, Dai N, Yu HG, Si JM. Curcumin reverses chemoresistance of human gastric cancer cells by downregulating the NF-kappaB transcription factor. Oncol Rep. 2011;26(5):1197–203. 10.3892/or.2011.1410 21811763

[28] Fu H, Wang C, Yang D, Wei Z, Xu J, Hu Z, et al. Curcumin regulates proliferation, autophagy, and apoptosis in gastric cancer cells by affecting PI3K and P53 signaling. J Cell Physiol. 2018;233(6):4634–42. 10.1002/jcp.26190 28926094

[29] Zheng X, Ma YF, Zhang XR, Li Y, Zhao HH, Han SG. circ_0056618 promoted cell proliferation, migration and angiogenesis through sponging with miR-206 and upregulating CXCR4 and VEGF-A in colorectal cancer. Eur Rev Med Pharmacol Sci. 2020;24(8):4190–202. 10.26355/eurrev_202004_20999 32373955

[30] Chen X, Su X, Zhu C, Zhou J. Knockdown of hsa_circ_0023028 inhibits cell proliferation, migration, and invasion in laryngeal cancer by sponging miR-194-5p. Biosci Rep. 2019;39(6):BSR20190177. 10.1042/BSR20190177 PMC6567676 31123169

[31] Gao Y, Wu P, Ma Y, Xue Y, Liu Y, Zheng J, et al. Circular RNA USP1 regulates the permeability of blood-tumour barrier via miR-194-5p/FLI1 axis. J Cell Mol Med. 2020;24(1):342–55. 10.1111/jcmm.14735 PMC6933377 31654502

[32] Tan X, Zhou C, Liang Y, Lai YF, Liang Y. circ_0001971 regulates oral squamous cell carcinoma progression and chemosensitivity by targeting miR-194/miR-204 in vitro and in vivo. Eur Rev Med Pharmacol Sci. 2020;24(5):2470–81. 10.26355/eurrev_202003_20515 32196598

[33] Bao J, Zou JH, Li CY, Zheng GQ. miR-194 inhibits gastric cancer cell proliferation and tumorigenesis by targeting KDM5B. Eur Rev Med Pharmacol Sci. 2016;20(21):4487–93. 27874950

[34] Wei R, Ding C, Rodriguez RA, Del Mar Requena Mullor M. The SOX2OT/miR-194-5p axis regulates cell proliferation and mobility of gastric cancer through suppressing epithelial-mesenchymal transition. Oncol Lett. 2018;16(5):6361–8. 10.3892/ol.2018.9433 PMC6202518 30405772

[35] Bahrami A, Ferns GA. Effect of curcumin and its derivates on gastric cancer: molecular mechanisms. Nutr Cancer. 2021;73(9):1553–69. 10.1080/01635581.2020.1808232 32814463

